# Involvement of ghrelin in the neonatal exposure to lipopolysaccharide and thermoregulation during endotoxemia in adulthood

**DOI:** 10.1186/cc12970

**Published:** 2013-11-05

**Authors:** Mayara Andrade Ferrari, Marcelo Eduardo Batalhão, Evelin Capellari Cárnio

**Affiliations:** 1 School of Nursing of Ribeirão Preto - University of São Paulo (USP), Ribeirão Preto, Brazil

## Background

Prior exposure to infection, particularly during the neonatal phase, contributes to individual differences in susceptibility to disease during adult life. Animal neonates undergoing lipopolysaccharide administration (LPS) react differently to the front endotoxemia in adulthood. Ghrelin, a peptide hormone originally found in the stomach, has effects on the modulation of the inflammatory response. Specific receptors are found for ghrelin on neutrophils, macrophages and lymphocytes and their activation by ghrelin inhibits the production of several inflammatory cytokines, including nitric oxide (NO). Therefore, our objective is to evaluate the role of ghrelin in the attenuation of fever during endotoxemia in adulthood induced by neonatal exposure to LPS.

## Materials and methods

The study was conducted using rats in the pregnancy period. After the birth of pups (day 0 of the experiment) we selected only male rats. All animals were weaned at 21 days and at 14 days of age received neonatal administration of LPS 100 μg/kg intraperitoneally (i.p.). Subsequently they were separate in cages until they reached 8 to 12 weeks of age for the experiment (by endotoxemia in adult administration of 10 mg/kg LPS i.p.). To determine the body temperature, the animals were anesthetized and a capsule inserted into the peritoneal cavity biotelemetry. Body temperature was measured for a period of 6 hours after induction of endotoxemia. To verify the effect of ghrelin and ghrelin antagonist on body temperature during endotoxemia, ghrelin was administered 0.1 mg/kg ghrelin antagonist or 50 nmol/kg i.p. concomitant administration of LPS. After decapitation, blood samples were collected and centrifuged to separate the plasma. The plasma was stored at -70°C for subsequent determination of NO.

## Results

In our preliminary data we observed no significant difference in fever-induced endotoxemia in animals subjected to LPS administration in the neonatal period, when compared with their respective controls.

## Conclusions

These data do not corroborate the findings of the literature and we believe it is due to the fact that the animals used until now have had prior exposure to pathogens. So in our next experiments we will use experimental animals that are specific pathogen free.

